# Modulating the Gut–Ovarian Axis: Mechanistic Rationale and the Limits of Current Evidence for Probiotics in Female Reproductive Aging

**DOI:** 10.1002/fsn3.72454

**Published:** 2026-10-06

**Authors:** Meiqi Chen, Qi Su, Mengshuang Sun, He Zhang, Jin Xu, Chenxi Zhang, Yanbo Wang

**Affiliations:** ^1^ College of Life Sciences and Food Engineering Inner Mongolia Minzu University Tongliao China; ^2^ Inner Mongolia Engineering and Technical Research Center for Personalized Medicine Tongliao China

**Keywords:** gut–ovarian axis, ovarian aging, ovarian reserve, premature ovarian insufficiency, probiotics

## Abstract

Female reproductive aging is characterized by progressive follicular depletion, deterioration of oocyte quality, and endocrine decline, with oxidative stress, mitochondrial dysfunction, immune‐inflammatory remodeling, and granulosa‐cell loss contributing to ovarian functional deterioration. This critical narrative review, supported by a structured literature search, evaluates mechanistic, preclinical, and human evidence for probiotic and microbiota‐targeted modulation of the gut–ovarian axis. The current evidence base remains predominantly preclinical. In premature ovarian insufficiency (POI), polycystic ovary syndrome (PCOS), chemotherapy‐induced ovarian injury, and reproductive‐aging models, selected interventions have modified intestinal microbial ecology, barrier function, microbial metabolites, and systemic immune–metabolic–endocrine signaling, with model‐specific effects on ovarian redox homeostasis, granulosa‐cell survival, follicular development, mitochondrial function, and oocyte‐related outcomes. Mechanistic support is strongest where gut dependency, metabolite supplementation, or related causal approaches have been demonstrated, but responses remain highly strain‐, model‐, dose‐, route‐, and host‐dependent. Human trials in PCOS and menopause‐related or reproductive settings have reported changes in selected metabolic, hormonal, menstrual, symptom, quality‐of‐life, and fertility‐related outcomes; however, our structured literature and trial‐registry searches did not identify a randomized human trial specifically designed to test whether probiotic or microbiota‐targeted intervention slows the longitudinal physiological loss of ovarian reserve or delays physiological ovarian aging. Findings from disease models, fecal microbiota transplantation, or other experimental interventions should therefore not be equated with the efficacy of oral probiotic foods or supplements. Current evidence supports biological plausibility and translational potential rather than established ovarian anti‐aging efficacy. Future studies should evaluate well‐characterized oral strains using prespecified ovarian‐aging‐relevant endpoints, mechanistic biomarkers, and standardized safety assessment.

## Graphical abstract

Selected orally administered probiotics and functional‐food interventions may alter gut microbial ecology and intestinal barrier function. Microbiota‐derived metabolites, microbial components, and endocrine signals—including short‐chain fatty acids, bile acids, tryptophan metabolites, and lipopolysaccharide—may influence systemic immune, metabolic, and hormonal pathways relevant to ovarian redox balance, mitochondrial function, inflammatory remodeling, granulosa‐cell survival, follicular reserve, steroidogenesis, and oocyte quality. In preclinical models of premature ovarian insufficiency, polycystic ovary syndrome, chemotherapy‐induced ovarian injury, and reproductive aging, selected microbiota‐targeted interventions have improved ovarian or reproductive outcomes, although effects are strain‐, model‐, and context‐dependent. Human studies in related reproductive and menopausal settings report selected metabolic, hormonal, menstrual, and symptom outcomes, but do not establish that probiotics slow physiological ovarian aging. Translation requires well‐characterized oral strains, clinically feasible regimens, prespecified longitudinal ovarian endpoints, mechanistic validation, and safety assessment.

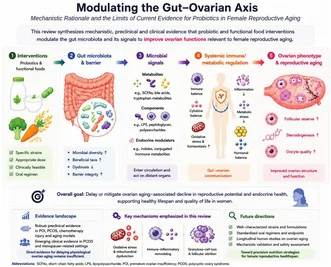

The ovary is central to female reproductive and endocrine health throughout the lifespan. Beyond its essential roles in follicular development, ovulation, and steroidogenesis, the age‐related loss of ovarian function is accompanied by progressive depletion of the follicular reserve, deterioration of oocyte quality, and endocrine decline, with consequences extending beyond fertility to broader aspects of women's health (Ahlborg et al. 2001; Galatidou et al. 2024; Hansen et al. 2008; Smits et al. 2023; Zhu et al. 2019). Ovarian aging is multifactorial, but three interconnected processes are particularly relevant to the gut–ovarian axis: oxidative stress and mitochondrial dysfunction, chronic immune‐inflammatory remodeling, and dysregulated cell death associated with follicular attrition. Excess reactive oxygen species and impaired antioxidant defenses can compromise oocytes and granulosa cells, while persistent inflammatory signaling can disturb the follicular microenvironment and steroidogenic function; these processes may converge on granulosa‐cell dysfunction and loss, thereby contributing to declining follicular integrity and ovarian function (Briley et al. 2016; Isola et al. 2024; Lim et al. 2015; Sadraie et al. 2000; Yao et al. 2024).

Because ovarian aging involves multiple interacting biological pathways, food‐derived bioactive compounds and microbiota‐modulating dietary components have attracted increasing interest as candidate multi‐target interventions. Experimental studies indicate that plant polyphenols and selected dietary fibers can modulate oxidative stress, inflammatory signaling, and gut microbial metabolism in models of ovarian dysfunction (Chen et al. 2022; Dong et al. 2026; Wu et al. 2019). Within this broader framework, probiotics represent a distinctive microbiota‐targeted strategy because they can act initially at the intestinal level while potentially influencing systemic metabolic, immune, and endocrine signaling. Appropriately selected probiotic strains may contribute to the maintenance of microbial homeostasis and intestinal barrier integrity and may modify the production of microbiota‐derived metabolites (Ahmadi et al. 2020; Alvarez et al. 2020). Their compatibility with oral and food‐based administration also supports their potential relevance to functional‐food and nutritional interventions. Nevertheless, probiotic effects are highly strain‐, dose‐, and host‐dependent, and their safety and biological efficacy should not be generalized across different probiotic preparations (Alvarez et al. 2020; Goh et al. 2026; Kekkonen et al. 2008).

Accumulating evidence supports bidirectional communication between the intestinal microbiota and ovarian/endocrine function, although both the direction and causality of these relationships remain context‐dependent (Chen et al. 2024; Qi et al. 2019; Zhao et al. 2022). Gut‐derived metabolites and microbial products may influence host and ovarian physiology through pathways involving short‐chain fatty acids (SCFAs) and G‐protein‐coupled receptors, lipopolysaccharide (LPS)–Toll‐like receptor 4 (TLR4)–nuclear factor‐κB (NF‐κB) signaling, bile acid–farnesoid X receptor (FXR) and G protein‐coupled bile acid receptor 1 (TGR5) signaling, tryptophan metabolism, and other immune–metabolic routes. Conversely, ovarian and endocrine status may alter host metabolism and the intestinal microbial environment, supporting a bidirectional rather than exclusively gut‐to‐ovary model. Conceptually, the available intervention evidence can be organized into an intestinal → systemic → ovarian sequence in which microbiota‐targeted interventions first alter microbial ecology, barrier function, or metabolic output and subsequently modify circulating immune, metabolic, and endocrine signals that may influence ovarian processes (Feng et al. 2024; Qi et al. 2019; Wang, Zeng, et al. 2026; Yao et al. 2026). Importantly, however, the evidence base remains predominantly preclinical. Direct mechanistic support is derived mainly from animal models of POI, chemotherapy‐induced ovarian injury, reproductive aging, or related ovarian disorders such as PCOS (Feng et al. 2024; Qi et al. 2019; Wang, Chen, et al. 2026; Yao et al. 2026). Human probiotic trials have been conducted in PCOS and several menopause‐related or reproductive settings (Honda et al. 2024; Lim et al. 2020; Rodríguez‐Palmero et al. 2026; Sun et al. 2026; Wu et al. 2026; Yin et al. 2026). However, our structured literature and trial‐registry searches did not identify a randomized human trial specifically designed to test whether probiotic or microbiota‐targeted intervention slows the longitudinal physiological loss of ovarian reserve. Registered studies in POI, DOR, and related ovarian‐dysfunction settings do include AMH, AFC, and other ovarian‐function outcomes, but these disease‐specific or short‐term endpoints are not equivalent to demonstrating a reduced rate of physiological age‐related follicular depletion. Disease‐model improvement, short‐term changes in reproductive‐endocrine biomarkers, and experimental microbiota transplantation should therefore not be interpreted as clinical proof that probiotics delay physiological ovarian aging in women.

Accordingly, this critical narrative review synthesizes mechanistic, preclinical, and human translational evidence concerning probiotic and microbiota‐targeted modulation of the gut–ovarian axis in female reproductive aging. Particular attention is given to oxidative stress and mitochondrial dysfunction, immune‐inflammatory remodeling, and granulosa‐cell loss/follicular attrition as ovarian processes with plausible interfaces to gut‐derived signals. Rather than treating all microbiota‐related findings as equivalent evidence, we critically evaluate studies according to the directness of their ovarian endpoints, experimental or clinical context, strain identity, dose, formulation, administration route, mechanistic validation, safety assessment, and human translatability. By distinguishing direct ovarian evidence from disease‐model evidence and mechanistic inference, this review aims to define both the current biological rationale and the evidentiary requirements for developing microbiota‐targeted functional‐food and precision‐nutrition strategies for female reproductive health.

## 1 Review Approach and Literature Search Strategy

The bibliographic searches covered database inception through September 18, 2026. We conducted a critical narrative review supported by a structured and auditable literature search. PubMed and the Web of Science Core Collection were searched using three complementary strategies in each database: a broad topic search, a focused human‐intervention/evidence‐gap search, and an anti‐Müllerian hormone (AMH)/antral follicle count (AFC) safety‐net search. Across the six searches, 4741 exported record occurrences were retrieved (2295 from PubMed and 2446 from Web of Science). Records were deduplicated sequentially by DOI, PMID, and exact title plus publication year, yielding 2763 unique bibliographic records that formed the structured source library for this narrative synthesis. The complete Boolean strings, search interfaces, search settings, record counts, deduplication procedures, record flow, and trial‐registry audit are provided in Data S1.

Study selection was guided by relevance to female reproductive aging and the gut–ovarian axis. Direct evidence included human or experimental studies of probiotics, synbiotics, fecal microbiota transplantation, or other microbiota‐targeted interventions reporting ovarian, follicular, oocyte, reproductive‐endocrine, fertility, or ovarian‐reserve outcomes. Studies outside an ovarian or reproductive context were retained only when they provided specific mechanistic information relevant to gut–ovarian communication and were explicitly interpreted as indirect evidence. For the focused human evidence‐gap assessment, studies in PCOS, premature ovarian insufficiency (POI), diminished ovarian reserve (DOR), menopause, or iatrogenic reproductive states were retained as disease‐ or context‐specific translational evidence but were not considered direct tests of slowed physiological ovarian aging unless the study design explicitly evaluated longitudinal ovarian‐reserve decline.

To evaluate the specific evidence gap concerning preservation of ovarian reserve, we additionally conducted a trial‐registry audit. ClinicalTrials.gov was searched using 17 prespecified intervention‐, condition‐, and outcome‐based queries, yielding 38 retrieval occurrences corresponding to 26 unique NCT records. Three complementary searches of the WHO International Clinical Trials Registry Platform (ICTRP) yielded 86 portal‐reported record occurrences, 85 exported trial rows, and 65 unique primary registry identifiers. After exact‐identifier linkage between ClinicalTrials.gov and WHO ICTRP, the combined provisional registry set comprised 75 unique study entries. A focused evidence‐gap assessment was then used to distinguish trials reporting ovarian‐reserve‐related outcomes from trials capable of testing longitudinal physiological ovarian‐reserve decline. Detailed registry queries, record linkage, publication checks, and the focused evidence‐gap screening are provided in Data S1.

Native searches of the Chinese Clinical Trial Registry (ChiCTR) were prespecified using 11 Chinese and 11 corresponding English keyword combinations. During the registry audit, however, the ChiCTR search interface returned an HTTP 405 security‐block response, preventing retrieval of query‐specific native search counts. ChiCTR records identified through WHO ICTRP were retained in the audit, but these records were not treated as equivalent to completion of the prespecified native ChiCTR searches. This limitation is explicitly documented in Data S1.

For the focused evidence‐gap verification, two authors independently screened the potentially relevant human intervention and registry records against the prespecified criteria, with disagreements resolved by consensus. The purpose of this structured search and registry audit was to improve the reproducibility and auditability of this critical narrative review; it was not intended to reclassify the review as a systematic review or meta‐analysis. The first online‐publication dates and bibliographic metadata of 2026 references were audited separately from the literature‐search record. This reference‐date audit is reported in Data S1 and did not retrospectively alter the archived bibliographic search‐flow counts.

For human intervention studies, we additionally extracted sample size, randomization, blinding, comparator, prospective trial registration, attrition, funding/sponsorship, and the directness of ovarian or reproductive endpoints. These design characteristics are summarized descriptively in Table S2 and were used to calibrate the confidence placed on individual human findings rather than to generate a formal numerical quality score. For the two recent PCOS meta‐analyses, primary‐study overlap was assessed at the unique trial/cohort level, and the magnitude of selected pooled effects and corresponding heterogeneity statistics are summarized in Table S3.

Evidence‐tier assignment was independently performed by two authors using the criteria defined in Table 1. Disagreements were resolved by consensus. Final classifications were cross‐checked for consistency with Table 1 and the terminology used throughout the manuscript.

**TABLE 1:** Preclinical and human translational evidence for probiotic or microbiota‐targeted interventions relevant to ovarian function and female reproductive aging.

Evidence tier	Strain/intervention	Model/population	Reproductive condition	Dose	Route	Duration	Ovarian/reproductive endpoints	Gut/metabolic mechanism tested	Main limitation	References
Tier A*	*Lactobacillus salivarius* Li01, CGMCC 7045	Female C57BL/6 mice; Tripterygium glycoside‐induced POI	Induced POI	200 μL of ~1 × 10^9^ CFU/mL/day (~2 × 10^8^ CFU/day)	Oral gavage	Daily during 35‐day protocol; POI induction during final 2 weeks	Improved estrous cyclicity and ovarian coefficient; normalized AMH/FSH and increased E2; preserved primary/developing follicles; reduced ovarian inflammatory injury and improved SOD/GPX4‐related antioxidant indices	Experimentally supported gut dependence: ovarian/endocrine protection was largely lost in germ‐free mice. Microbiome and metabolomic analyses were associated with alterations in tryptophan–kynurenine metabolism and ovarian NAD^+^‐related pathways; NAMPT/CD38/SIRT1 changes were also associated with the intervention response.	Chemically induced POI rather than physiological aging; largely preventive/co‐treatment design. The complete Li01→kynurenine→NAD^+^ pathway is not equally demonstrated at every step, and several downstream effects were strongest with Li01 plus tryptophan	(Yao et al. 2026); PMID 41951629; DOI https://doi.org/10.1038/s41538‐026‐00823‐6
Tier A*	Limosilactobacillus reuteri DSM 17938; microbiota‐associated metabolite β‐resorcylic acid (β‐RA)	Female mice; cisplatin‐induced ovarian toxicity/POI	Chemotherapy‐induced POI	Limosilactobacillus reuteri: 1 × 10^9^ CFU/day	Oral gavage	Began 3 days before cisplatin and continued during cisplatin modeling	Increased ovarian weight; improved E2/FSH; preserved primordial, growing and antral follicles; reduced follicular/granulosa‐cell apoptosis. β‐RA experiments additionally supported oocyte, embryo‐development and fertility‐related protection	Limosilactobacillus reuteri was linked to restoration/increased availability of β‐RA. Mechanistic validation was strongest for β‐RA, which reduced nuclear SOX7 accumulation and downstream BAX‐associated granulosa‐cell apoptosis	Chemotherapy injury rather than physiological aging; preventive exposure. The downstream SOX7/BAX mechanism was demonstrated primarily with β‐RA rather than with the live strain itself.	(Feng et al. 2024); PMID 39096912; DOI https://doi.org/10.1016/j.xcrm.2024.101678
Tier A*	Young‐donor FMT, with downstream Bacteroides caecimuris and glutamate experiments	Young 6–8‐week and advanced reproductive‐age 50–58‐week female ICR mice	Advanced reproductive aging	FMT: 100 mg feces/mL suspension, 200 μL/gavage (~20 mg fecal material/dose); not CFU‐standardized	Oral gavage	FMT on days 4–8 and day 15 after antibiotic conditioning; downstream glutamate and bacterial experiments had separate schedules	Improved ovulation, number/quality of ovulated oocytes, polar‐body extrusion, spindle/chromosomal integrity and fertility/litter‐related outcomes in aged recipients	Multi‐omics analyses associated microbiota changes with ovarian glutamate. Separate glutamate administration and complementary Bacteroides caecimuris experiments experimentally supported components of the proposed microbiota–glutamate pathway, including improvement in mitochondrial/redox and reproductive phenotypes	Mouse‐only; FMT benefits diminished substantially by ~30 days; authors note that the detailed mechanistic connection between Bacteroides caecimuris and ovarian glutamate remains incompletely resolved	(Wang, Zeng, et al. 2026); PMID 42103932; DOI https://doi.org/10.1038/s44321‐026‐00443‐3
Tier A	Young‐donor fecal microbiota transplantation (FMT)	42‐week‐old female mice receiving microbiota from 5‐week‐old female donors	Reproductive aging	100 μL fecal supernatant per gavage; not CFU‐standardized	Oral FMT	Three times weekly for 8 weeks after 2 weeks of antibiotic conditioning	Increased first‐litter size; reduced follicular atresia/apoptosis; increased ovarian‐cell proliferation; decreased ovarian macrophage accumulation and macrophage‐derived multinucleated giant cells	Shifted recipients toward a younger‐donor‐associated microbiota, including increased Bifidobacterium/Ruminococcaceae‐related signals; systemic IL‐4 increased and IFN‐γ decreased	FMT is an undefined microbial consortium, not a probiotic strain; individual organisms/metabolites cannot be assigned causality; antibiotic/FMT procedures are not equivalent to food supplementation	(Xu et al. 2022); DOI https://doi.org/10.1016/j.jgg.2022.05.006; PMID 35654347
Tier B	*Akkermansia muciniphila* PROBIO (CGMCC 20955)	Female Sprague–Dawley rats; DHEA‐induced PCOS	PCOS	1 × 10^9^ CFU/mL preparation; the administered volume was not clearly reported in the primary Methods; therefore, CFU/day could not be determined	Oral administration/gavage	35 days: 2‐week pretreatment followed by continuation during 3‐week DHEA induction	Reduced androgen‐associated abnormalities and LH/FSH; restored estrous cyclicity; reduced cystic follicles; increased corpora lutea and improved granulosa‐layer morphology	16S rRNA gene sequencing and metabolomic analyses associated the intervention with enhanced arginine biosynthesis and L‐arginine availability; separate L‐arginine supplementation experimentally supported mediator involvement by reproducing several reproductive improvements	DHEA‐PCOS, not ovarian aging/POI; partly prophylactic design; metabolite‐rescue evidence strengthens but does not fully establish every step of an *Akkermansia muciniphila* →arginine→ovary causal chain	(Wu et al. 2025); PMID 41324840; DOI https://doi.org/10.1007/s11684‐025‐1161‐3
Tier B	*Bifidobacterium animalis* subsp. lactis BGI‐L99	DHEA‐induced PCOS mice	PCOS	1 × 10^9^ CFU in 200 μL/day	Oral gavage	21 days after PCOS induction	Restored estrous cyclicity; reduced cystic follicles and increased corpora lutea; lowered testosterone and increased E2; ovarian steroidogenic/receptor markers were altered	Microbiota remodeling accompanied restoration of CDCA/TUDCA and activation of TGR5/GATA3/IL‐22‐related signaling. Separate CDCA/TUDCA treatment reproduced several reproductive phenotypes and increased IL‐22, providing relatively strong mediator‐level evidence	PCOS rather than ovarian aging/POI; bile‐acid rescue does not constitute definitive proof of the complete BGI‐L99 → TGR5 → IL‐22 causal chain; genetic knockout validation remains lacking; industry affiliations should be acknowledged when interpreting the study	(Wang, Chen, et al. 2026); DOI https://doi.org/10.1002/imm3.70047
Tier B‐human	*Bifidobacterium lactis* V9	14 women with PCOS completing the probiotic phase of a two‐stage cohort study	PCOS	10.6 log CFU once daily	Oral	10 weeks	LH and LH/FSH decreased in 9 apparent responders; endocrine response was heterogeneous across participants	Viable V9 colonization correlated with clinical/SCFA response; intervention altered intestinal microbiota and SCFAs. Authors proposed an SCFA→PYY/ghrelin→hypothalamic–pituitary sex‐hormone pathway	Very small, uncontrolled intervention cohort; only 14 completed without PCOS medication; 9/14 vs. 5/14 response heterogeneity; no follicular‐reserve, ovarian morphology or fertility endpoint; proposed gut–brain mechanism is primarily correlational	(Zhang et al. 2019); PMID 31020040; DOI https://doi.org/10.1128/msystems.00017‐19
Tier B‐human	Seven‐strain probiotic + FOS: *Lactobacillus acidophilus* UBLA‐34, Lacticaseibacillus rhamnosus UBLR‐58, Limosilactobacillus reuteri UBLRu‐87, Lactiplantibacillus plantarum UBLP‐40, Lacticaseibacillus casei UBLC‐42, Limosilactobacillus fermentum UBLF‐31, *Bifidobacterium bifidum* UBBB‐55	104 women aged 18–40 years with PCOS; randomized placebo‐controlled intervention with standardized lifestyle advice	PCOS	Formulation contained 1 × 10^10^ CFU in total plus 100 mg FOS per stated capsule/formulation	Oral capsule	6 months	Improved menstrual‐cycle regularity; total testosterone decreased; ovarian ultrasound and reproductive‐endocrine variables were assessed	Plasma LPS and metabolic/inflammatory variables were examined, supporting a possible intestinal/systemic component, but a strain‐specific ovarian mechanism was not established	Multi‐strain formulation prevents attribution to a single strain; concomitant lifestyle intervention; PCOS rather than ovarian aging; no demonstration of slowed follicular‐reserve loss	(Kaur et al. 2022); PMID 35857132; DOI https://doi.org/10.1007/s00394‐022‐02959‐z
Tier B‐human	Probiotic preparation; strain composition and viable count were not sufficiently specified in the accessible publication record	60 non‐obese women with PCOS randomized to probiotics, metformin, or combination treatment	PCOS	4 g/day probiotic preparation	Oral	12 weeks	Menstrual recovery occurred in 40% with probiotics, 55% with metformin and 80% with combination treatment; ovulation rates were 30%, 55% and 75%, respectively; AMH, testosterone and FAI also changed	Mechanism not directly established; study principally compared clinical/endocrine outcomes rather than demonstrating a specific gut‐metabolite‐ovary pathway	Small sample; no true placebo group; probiotic formulation is insufficiently transparent in the readily accessible primary record; combination arm prevents attribution of combined effect to probiotics	(Ji et al. 2022); PMID 36062569; DOI https://doi.org/10.1080/09513590.2022.2119219
Tier B‐human	Traditional grain kefir, a complex fermented‐food consortium rather than a defined strain	17 women with PCOS; uncontrolled before–after intervention	PCOS, but no direct ovarian endpoint	250 mL/day	Oral fermented beverage	8 weeks	No direct ovarian‐reserve, ovarian‐morphology, ovulation or fertility outcome was evaluated	IL‐6 decreased; gut‐community changes included increased Bacilli/Lactococcus‐related signals and reduced Holdemania; zonulin did not significantly improve	Very small sample; no placebo/control group; complex food matrix and multiple bacteria/yeasts prevent strain attribution. No direct ovarian‐aging endpoint was assessed; therefore, this study does not provide evidence that kefir delays ovarian aging.	(Çıtar Dazıroğlu et al. 2024); PMID 39139979; DOI https://doi.org/10.1002/fsn3.4212
Tier B‐human	*Lactobacillus acidophilus* YT1 (MENOLACTO; KCCM11808P)	Women aged 40–60 years with menopausal symptoms; multicenter, randomized, double‐blind, placebo‐controlled trial	Menopausal symptoms/perimenopausal–menopausal population	1 × 10^8^ CFU/day	Oral dietary supplement	12 weeks	Significantly improved total Kupperman Menopausal Index and menopause‐specific quality‐of‐life scores; no significant between‐group changes in serum FSH or estradiol (E2); no significant change in endometrial thickness	A specific gut–ovarian mechanism was not directly demonstrated in this clinical trial; the study primarily evaluated symptomatic efficacy and safety	Symptom‐oriented menopause trial rather than ovarian‐aging efficacy study; no AMH, AFC, follicular reserve, ovulation, oocyte‐quality, or rate‐of‐ovarian‐aging endpoint; symptom improvement should not be interpreted as preservation of ovarian function	(Lim et al. 2020); DOI https://doi.org/10.3390/jcm9072173; PMID 32660010
Tier B‐human	Levilactobacillus brevis KABP052 + Lactiplantibacillus plantarum KABP051 + *Pediococcus acidilactici* KABP021	Healthy Japanese women aged 45–55 in peri−/postmenopausal transition; randomized, double‐blind, placebo‐controlled trial; 115 randomized, 111 in per‐protocol analysis	Menopausal transition	≥ 1 × 10^9^ CFU/day; strains 2:1:1	Oral capsule	12 weeks	E1 and E2 maintained/higher relative to placebo after adjustment; no significant between‐group improvement in mKMI symptoms	KABP052 showed estrogen‐deconjugating β‐glucuronidase activity in vitro; fecal GUS activity did not differ significantly between groups; gut microbiota not profiled	No AMH, AFC, follicular‐reserve, ovulatory, or ovarian‐aging‐rate endpoint; exploratory study; no demonstrated association between estrogen maintenance and symptom improvement; industry‐funded; same KABP052/KABP051/KABP021 formulation and overlapping industry‐affiliated investigators as Rodríguez‐Palmero et al. (2026), and therefore not an independent product replication	(Honda et al. 2024); DOI https://doi.org/10.1089/jmf.2023.k.0320
Tier B‐human	Limosilactobacillus reuteri NCU‐37	Infertile women aged 20–40 with leuprorelin‐induced perimenopausal syndrome; 100 LIPS patients randomized to probiotic/placebo plus 50 healthy reference participants	Leuprorelin‐induced perimenopausal syndrome during ART	2 × 10^9^ CFU/sachet, twice daily = 4 × 10^9^ CFU/day	Oral sachet	4 weeks	MKI, anxiety/depression/insomnia scores improved; AMH, E2, FSH and LH higher vs. placebo; pregnancy rate numerically 72.3% vs. 65.2%, with statistical significance for pregnancy difference not clearly reported in the main result	Fecal microbiota composition/diversity changed; Lactobacillus increased, Bacteroides and Prevotella decreased; Akkermansia enriched in differential analysis	Pharmacologically induced reversible HPO suppression, not physiological perimenopause or POI; short intervention; AMH change does not demonstrate slowed follicular depletion	(Wu et al. 2026); DOI https://doi.org/10.1039/D5FO04330A
Tier B‐human	Levilactobacillus brevis KABP052 + Lactiplantibacillus plantarum KABP051 + *Pediococcus acidilactici* KABP021	245 women aged 45–60 in perimenopause/postmenopause; randomized, double‐blind, placebo‐controlled real‐world trial	Menopause‐related symptoms	1 × 10^9^ CFU/day	Oral capsule	3 months	Menopause Rating Scale II total score and somato‐vegetative domain improved; Utian QoL improved; overall Cervantes QoL not significantly different	No microbiome or mechanistic biomarker assessment in this trial	Symptom/QoL study with no circulating sex‐hormone, AMH, AFC, follicular‐reserve, or ovarian‐aging‐rate endpoint; industry‐funded with sponsor‐affiliated investigators; same KABP052/KABP051/KABP021 formulation and overlapping investigators as Honda et al. (2024), and therefore not an independent product replication.	(Rodríguez‐Palmero et al. 2026); DOI https://doi.org/10.1097/GME.0000000000002871
Tier C	Estropausal‐donor FMT	Young adult female mice receiving FMT from young or 20‐month‐old estropausal donors	Ovarian‐aging‐associated microbiome experiment	Donor fecal suspension 20 mg/mL; 100 μL/recipient/dose (~2 mg fecal material)	Oral gavage after microbiota depletion	Reported as biweekly for 3 weeks	Unexpectedly, estropausal‐donor FMT improved a composite ovarian‐health phenotype, increased follicle‐related measures and altered ovarian inflammatory/transcriptomic indices; latency to first litter decreased, whereas litter‐size improvement was not significant	Metagenomics, metabolomics and mediation analyses identified candidate taxa/metabolic pathways potentially linking the estropausal microbiome to ovarian outcomes	Recipients were young adults, not aged females, so this informs ovarian‐aging‐associated biology but not evidence that FMT rejuvenates an aged ovary. Candidate microbial mediators are largely inferred and require intervention/rescue validation	(Kim et al. 2026); PMID 41776310; DOI https://doi.org/10.1038/s43587‐026‐01069‐3

*Note:* Evidence‐tier definitions: Evidence tiers were developed for this narrative review to indicate the proximity and directness of evidence to ovarian aging/function and do not represent a formal GRADE assessment or an ordinal ranking of study quality or efficacy. Tier A denotes microbiota‐targeted intervention studies in physiological/reproductive‐aging or POI/ovarian‐insufficiency models with at least one direct ovarian, follicular, oocyte, ovulatory, ovarian‐reserve, or reproductive‐function endpoint. Tier A* denotes studies meeting Tier A criteria that additionally provide causal mechanistic validation of gut dependence or involvement of a gut‐derived mediator, for example through germ‐free or microbiota‐depletion experiments, defined‐metabolite supplementation/rescue/blockade, receptor/genetic intervention, or complementary microbial–metabolite intervention experiments. The asterisk indicates additional mechanistic validation and does not denote a higher level of clinical evidence or greater treatment efficacy. Tier B denotes disease‐context preclinical evidence, principally from PCOS or related ovarian‐dysfunction models not equivalent to physiological ovarian aging. Tier B‐human denotes human translational intervention evidence from PCOS, POI/DOR‐adjacent, peri−/postmenopausal, or iatrogenic reproductive settings; unless longitudinal physiological ovarian‐reserve decline was directly evaluated, these studies should not be interpreted as demonstrating slowed physiological ovarian aging. Tier C denotes mechanistic, boundary‐setting, or indirect evidence that informs biological plausibility, causal direction, or evidentiary limits but is more distal from physiological ovarian‐aging efficacy. Mechanistic interpretation: For evidence‐status labeling in Table 1 and Figure 2, four terms are used consistently: experimentally supported, associated, proposed, and inferred. “Experimentally supported” denotes relationships directly tested by intervention or mechanistic perturbation in the cited model. “Associated” denotes observed relationships without direct causal validation. “Proposed” denotes biologically plausible pathways suggested by the available evidence but not directly tested as complete mechanisms. “Inferred” denotes relationships derived indirectly from multi‐omic, mediation, or cross‐study evidence rather than demonstrated by direct causal intervention. Dose reporting: Doses are reported in the form used in the original publications. Concentration‐based preparations (e.g., CFU/mL) were not converted to CFU/day unless the administered volume was explicitly reported. FMT doses are not expressed as equivalent probiotic CFU because fecal microbial communities are complex, non‐standardized consortia.

Abbreviations: 16S, ribosomal RNA; 16S, rRNA; AFC, antral follicle count; AMH, anti‐Müllerian hormone; ART, assisted reproductive treatment; BAX, BCL2‐associated X protein; CD38, cluster of differentiation 38; CDCA, chenodeoxycholic acid; CFU, colony‐forming units; DHEA, dehydroepiandrosterone; E1, estrone; E2, estradiol; FAI, free androgen index; FMT, fecal microbiota transplantation; FOS, fructooligosaccharides; FSH, follicle‐stimulating hormone; GATA3, GATA‐binding protein 3; GPX4, glutathione peroxidase 4; GUS, β‐glucuronidase; HPO, hypothalamic–pituitary–ovarian; IFN‐γ, interferon gamma; IL, interleukin; LH, luteinizing hormone; LIPS, leuprorelin‐induced perimenopausal syndrome; LPS, lipopolysaccharide; MKI, modified Kupperman Index; mKMI, modified Kupperman Menopausal Index; NAD^+^, nicotinamide adenine dinucleotide; NAMPT, nicotinamide phosphoribosyltransferase; PCOS, polycystic ovary syndrome; POI, premature ovarian insufficiency; PYY, peptide YY; QoL, quality of life; SCFAs, short‐chain fatty acids; SIRT1, sirtuin 1; SOD, superoxide dismutase; SOX7, SRY‐box transcription factor 7; T, testosterone; TGR5, G protein‐coupled bile acid receptor 1; TUDCA, tauroursodeoxycholic acid; β‐RA, β‐resorcylic acid.

## 2 Core Mechanisms of Ovarian Aging Relevant to the Gut–Ovarian Axis

Ovarian aging results from interacting biological processes rather than a single molecular defect. For the purpose of this review, three processes are particularly relevant to gut–ovarian communication: oxidative stress and mitochondrial dysfunction, chronic immune‐inflammatory remodeling of the ovarian microenvironment, and dysregulated granulosa‐cell death associated with follicular reserve depletion. These mechanisms are closely interconnected, because mitochondrial dysfunction can amplify reactive oxygen species (ROS) production, inflammatory signaling can further disturb ovarian redox homeostasis, and both processes can compromise granulosa‐cell survival and follicular integrity. Accordingly, this section focuses on these ovarian processes as plausible downstream targets of gut‐derived metabolic and inflammatory signals, rather than providing an exhaustive overview of all mechanisms or therapeutic strategies involved in ovarian aging. These three interconnected ovarian processes and their proposed interfaces with gut‐derived metabolic, inflammatory, and endocrine signals are summarized in Figure 1.

**FIGURE 1:**
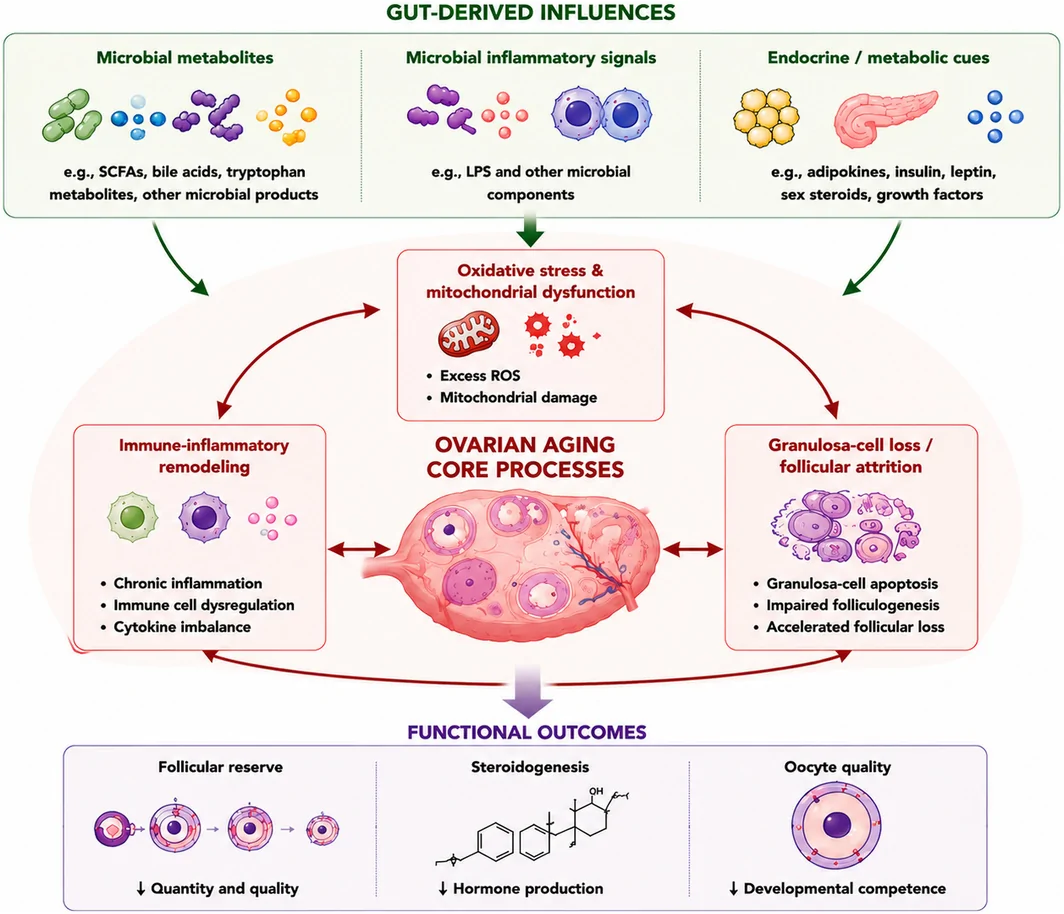
Conceptual framework of ovarian processes relevant to gut–ovarian communication during female reproductive aging. Gut‐derived metabolic, inflammatory, and endocrine signals may converge on three interconnected ovarian processes emphasized in this review: oxidative stress and mitochondrial dysfunction, immune‐inflammatory remodeling, and granulosa‐cell loss with follicular attrition. These processes may collectively influence follicular reserve, steroidogenesis, and oocyte quality. The figure provides a conceptual overview; specific microbiota‐derived pathways and their evidence levels are detailed in Figure 2. LPS, lipopolysaccharide; ROS, reactive oxygen species; SCFAs, short‐chain fatty acids.

### 2.1 Oxidative Stress and Mitochondrial Dysfunction

Oxidative stress and mitochondrial dysfunction are closely associated with the age‐related deterioration of ovarian function. Because oocytes and granulosa cells depend heavily on mitochondrial metabolism to support follicular growth, steroidogenesis, and oocyte competence, disruption of redox homeostasis can affect both the cellular environment of the follicle and the quality of the oocyte. In human ovarian tissue, advanced maternal age has been associated with increased oxidative damage and metabolic alterations consistent with impaired mitochondrial function, including age‐related changes in pathways related to nicotinamide, purine, and glutathione metabolism. These findings support the relevance of redox and mitochondrial abnormalities to physiological human ovarian aging rather than only to experimentally induced ovarian injury (Smits et al. 2023).

Experimental models further suggest that impaired antioxidant defense can contribute to follicular loss rather than merely accompany it. In mice lacking the glutamate cysteine ligase modifier subunit (Gclm), ovarian glutathione concentrations are markedly reduced, ovarian oxidative stress is increased, and age‐related depletion of the primordial follicle pool is accelerated. Increased granulosa‐cell apoptosis is also observed in later‐stage follicles, linking oxidative imbalance to follicular‐cell survival and reproductive decline (Lim et al. 2015). Collectively, these findings support a model in which age‐associated redox imbalance and mitochondrial dysfunction can weaken ovarian cellular resilience and increase susceptibility to follicular dysfunction and loss.

Why this matters to the gut–ovarian axis. The ovarian redox environment is potentially responsive to circulating metabolites generated or modified by the gut microbiota. Experimental work has shown that the microbial metabolite butyrate can signal through G protein‐coupled receptors 41 and 43 (GPR41/GPR43) in ovarian granulosa‐cell systems and influence mitochondrial and steroidogenic pathways, whereas more recent animal studies have linked microbiota‐derived short‐chain fatty acids (SCFAs) to follicular maturation through a GPR43‐dependent peri‐ovarian adipose–ovary pathway. These findings provide a mechanistic interface between gut microbial metabolism and ovarian redox/metabolic function, although direct evidence that this pathway delays physiological ovarian aging in humans is not yet available (Xu et al. 2025; Ye et al. 2021).

### 2.2 Immune Inflammation and Ovarian Microenvironment

Reproductive aging is accompanied by remodeling of the ovarian immune and stromal microenvironment. In reproductively aged mice, increased stromal collagen deposition, macrophage‐associated changes, and elevated inflammatory signals have been reported, suggesting that chronic low‐grade inflammation can alter the tissue environment in which follicles develop (Briley et al. 2016). More recent single‐cell profiling of the aging mouse ovary has further demonstrated age‐related shifts in immune‐cell abundance and in inflammatory, stress‐related, and extracellular‐matrix signaling across ovarian cell populations, including granulosa, theca, stromal, and immune cells (Isola et al. 2024).

Importantly, the inflammatory phenotype should not be equated automatically with universal ovarian fibrosis. In a longitudinal comparison of 2‐, 6‐, 12‐, and 18‐month‐old C57BL/6 mice, ovarian follicle depletion was accompanied by increased proportions of CD4+ T cells, B cells, and macrophages and by age‐related increases in several inflammatory mediators, including interleukin‐1α/β (IL‐1α/β), tumor necrosis factor‐α (TNF‐α), interleukin‐6 (IL‐6), apoptosis‐associated speck‐like protein containing a CARD (ASC), and NOD‐, LRR‐ and pyrin domain‐containing protein 3 (NLRP3). However, collagen I/III staining did not increase significantly with age in that cohort. Thus, ovarian inflammaging is supported by several lines of evidence, whereas the extent and timing of fibrotic remodeling appear to depend on model, age, and experimental context (Lliberos et al. 2021).

Evidence from human ovarian tissue also points to age‐associated immune remodeling, although the available datasets remain smaller than those in animal studies. Single‐cell analyses have identified age‐related changes in ovarian macrophage populations and inflammatory cell–stromal interactions, including signatures consistent with macrophage pyroptosis and stromal senescence. Experimental manipulation of pyroptotic macrophage signaling in complementary models further supports a possible contribution of inflammatory immune‐cell states to ovarian tissue aging (Zhou et al. 2024). These findings should nevertheless be interpreted as emerging mechanistic evidence rather than proof that a single inflammatory pathway drives human ovarian aging.

Why this matters to the gut–ovarian axis. Intestinal barrier integrity, microbial products, and microbiota‐derived metabolites can alter systemic immune signaling and therefore provide a plausible route by which gut perturbations may influence the ovarian inflammatory microenvironment. Importantly, direct mechanistic support is currently strongest in ovarian‐dysfunction models rather than in physiological human aging. For example, gut microbiota–bile acid–interleukin‐22 (IL‐22) signaling has been experimentally linked to ovarian dysfunction in PCOS, while a 2026 POI mouse study found that the ovarian anti‐inflammatory and antioxidant effects of *Lactobacillus salivarius* Li01 were lost under germ‐free conditions, supporting dependence on an intact gut microbial ecosystem. These studies establish biological plausibility for gut‐to‐ovary immune communication but should not be interpreted as direct clinical evidence of delayed ovarian aging (Qi et al. 2019; Yao et al. 2026).

### 2.3 Granulosa‐Cell Apoptosis and Follicular Reserve Depletion

Progressive depletion of the follicular reserve is a defining feature of reproductive aging, and granulosa‐cell survival is closely linked to the fate and functional competence of growing follicles. Granulosa cells provide metabolic, endocrine, and paracrine support to the developing oocyte; excessive loss or dysfunction of these cells can therefore contribute to follicular atresia and impaired follicular development. Human evidence is consistent with an age‐related increase in granulosa‐cell apoptosis: in women undergoing controlled ovarian stimulation for in vitro fertilization (IVF), older age was associated with a higher incidence of apoptotic granulosa cells and with lower numbers of retrieved and mature oocytes (Sadraie et al. 2000). Because these observations were obtained in a relatively small assisted‐reproduction cohort, they support an association with reproductive age but should not be treated as a population‐level causal demonstration.

Experimental ovarian‐aging studies provide additional mechanistic support for links among mitochondrial stress, cell death, and follicular decline. For example, reproductive aging in mice has been associated with impaired mitophagy and increased ovarian apoptosis, whereas manipulation of the mitophagy‐associated regulator Nur77 improved mitochondrial quality, reduced apoptotic signaling, and improved ovarian functional indices in aged mice (Yao et al. 2024). Likewise, the accelerated follicle depletion observed in antioxidant‐deficient Gclm‐null mice is accompanied by increased granulosa‐cell apoptosis, illustrating how oxidative stress and cell‐death pathways can converge during ovarian functional decline (Lim et al. 2015).

Follicular reserve depletion should nevertheless not be reduced to apoptosis alone. Age‐related loss of reproductive function reflects changes in primordial‐follicle activation and attrition, granulosa‐ and oocyte‐cell quality, mitochondrial competence, and the surrounding endocrine and stromal environment. Apoptosis is therefore best considered one component of an interconnected aging phenotype rather than the sole cause of declining ovarian reserve. This distinction is important when interpreting intervention studies in which reductions in apoptotic markers are sometimes described too broadly as evidence that “ovarian aging has been reversed.”

Why this matters to the gut–ovarian axis. Among the proposed probiotic mechanisms, protection of granulosa‐cell survival is one of the areas in which direct ovarian endpoints are beginning to emerge in preclinical disease models. In a cisplatin‐induced POI mouse model, oral supplementation with Limosilactobacillus reuteri DSM 17938 or its associated microbial metabolite β‐resorcylic acid (β‐RA) preserved follicular reserve and attenuated ovarian damage. Mechanistic experiments further showed that β‐RA reduced granulosa‐cell apoptosis by suppressing nuclear SRY‐box transcription factor 7 (SOX7) accumulation and downstream BAX‐mediated apoptotic signaling (Feng et al. 2024). Similarly, Li01 improved follicular and ovarian biochemical endpoints in a POI mouse model, with its effects abolished in germ‐free animals. These studies provide stronger gut–ovarian evidence than non‐ovarian anti‐apoptotic studies, but they remain models of induced ovarian insufficiency and cannot establish efficacy against physiological ovarian aging in women (Yao et al. 2026).

Together, these three interconnected ovarian processes provide the mechanistic framework for evaluating whether probiotic and microbiota‐targeted interventions can influence reproductive aging through gut‐derived metabolic, inflammatory, and endocrine signals. The following sections therefore distinguish direct ovarian evidence from mechanistic inference and from evidence obtained in related reproductive‐disorder models.

## 3 Probiotic and Microbiota‐Targeted Evidence Relevant to Ovarian Function

Evidence supporting probiotic and microbiota‐targeted interventions relevant to ovarian function varies substantially in its proximity to physiological ovarian aging, the directness of ovarian or reproductive outcomes, and the degree of mechanistic validation. Table 1 therefore uses an evidence architecture that distinguishes direct ovarian evidence from disease‐context, human translational, and mechanistic or boundary‐setting evidence. Tier A denotes direct ovarian evidence from physiological/reproductive‐aging or POI/ovarian‐insufficiency models, whereas Tier A* denotes Tier A evidence with additional experimental validation of gut dependence or involvement of a defined gut‐derived mediator. Tier B denotes disease‐context preclinical evidence, principally from PCOS or related ovarian dysfunctions, whereas Tier B‐human denotes human translational intervention evidence that does not establish slowed physiological ovarian aging. Tier C denotes mechanistic, indirect, or boundary‐setting evidence that informs biological plausibility or causal direction but is more distal from physiological ovarian‐aging efficacy. These categories indicate evidentiary proximity and mechanistic resolution rather than an ordinal ranking of overall study quality or treatment efficacy.

The evidence summarized in Table 1 spans different levels of proximity to physiological ovarian aging and different degrees of mechanistic validation. Direct ovarian evidence remains predominantly preclinical, while the available human intervention literature is largely translational and has not directly tested longitudinal physiological ovarian‐reserve decline. Direct defined‐strain probiotic evidence relevant to POI or reproductive aging remains predominantly preclinical. Among the most directly relevant interventions, *Lactobacillus salivarius* Li01 improved ovarian, endocrine, and follicular outcomes in an induced POI model, with loss of major protective effects under germ‐free conditions supporting dependence on an intact gut microbial ecosystem. Limosilactobacillus reuteri DSM 17938 likewise attenuated ovarian injury and preserved follicular and endocrine endpoints in a cisplatin‐induced model, whereas β‐resorcylic acid provided more direct mechanistic evidence linking the intervention to reduced SOX7/BAX‐associated granulosa‐cell apoptosis. Fecal microbiota transplantation (FMT) studies provide complementary evidence that manipulation of the intestinal microbial ecosystem can modify ovarian, oocyte, and fertility‐related phenotypes, although FMT should not be equated with a defined‐strain probiotic intervention. Human intervention evidence remains limited and is concentrated mainly in PCOS, where studies have predominantly assessed reproductive‐endocrine, menstrual, ovulatory, metabolic, or inflammatory outcomes rather than direct measures of physiological ovarian aging. A randomized trial of *Lactobacillus acidophilus* YT1 in women with menopausal symptoms further supports the feasibility of oral probiotic intervention in a menopause‐related population; however, the reported benefits were confined primarily to symptom and quality‐of‐life outcomes, whereas FSH and estradiol were not significantly altered (Lim et al. 2020). This study therefore provides translational evidence relevant to menopause‐related symptoms, but not evidence that probiotic supplementation preserves ovarian reserve or delays physiological ovarian aging. PCOS studies should likewise be regarded as evidence from a related ovarian dysfunction and should not be extrapolated as evidence that probiotics delay physiological ovarian aging. Accordingly, current findings support the biological plausibility that selected probiotic or microbiota‐targeted interventions may modulate gut–metabolic–immune pathways relevant to ovarian function, but they do not establish that probiotics delay physiological ovarian aging in women. Considerable heterogeneity in strain identity, formulation, dose, model, administration schedule, and endpoint selection further limits direct cross‐study comparison.

## 4 Mechanistic Framework of the Gut–Ovarian Axis: From Intestinal Modulation to Ovarian Responses

The evidence‐calibrated framework developed in this review is summarized in Figure 2, which integrates microbiota‐targeted interventions with gut‐local effects, microbial molecular relays, systemic immune–metabolic–endocrine signaling, and downstream ovarian outcomes. The following subsections examine each layer of this framework and distinguish experimentally supported relationships from proposed or incompletely validated mechanisms.

**FIGURE 2:**
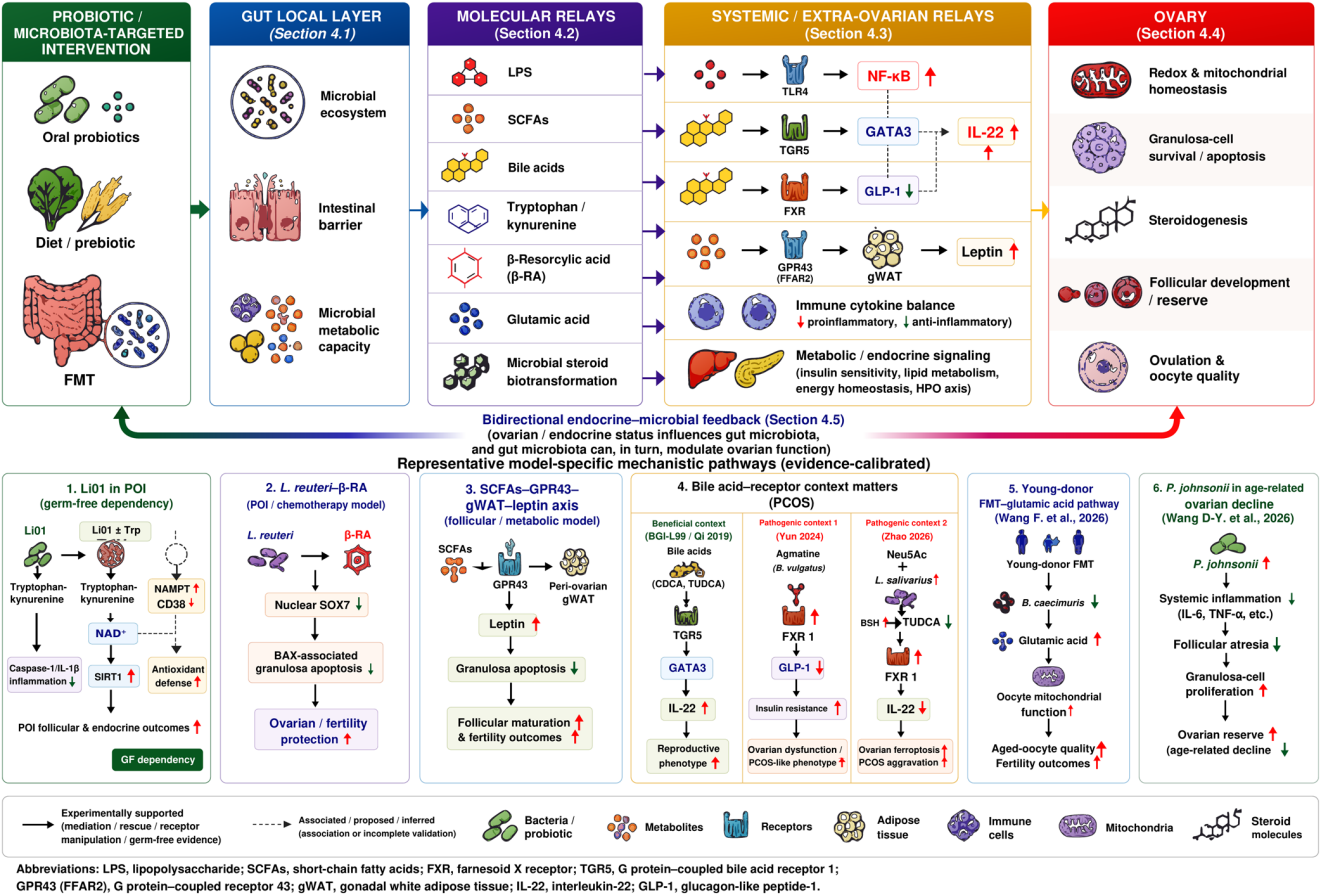
Evidence‐calibrated gut–systemic–ovarian signaling framework for probiotic and microbiota‐targeted modulation of ovarian function. The figure is read from left to right, from microbiota‐targeted interventions and gut‐local effects to microbial molecular relays, systemic immune–metabolic–endocrine signaling, and ovarian outcomes. Solid arrows indicate experimentally supported relationships demonstrated by direct intervention or mechanistic perturbation in the cited model(s). Dashed arrows indicate associated, proposed, or inferred relationships that have not been established by direct causal intervention in the relevant ovarian or reproductive context. FXR‐ and TGR5‐related pathways are shown separately to reflect their context‐dependent effects. The reverse arrow denotes bidirectional ovarian/endocrine–microbial communication. Evidence from POI, PCOS, chemotherapy‐induced ovarian injury, and other experimental models should not be interpreted as proof that these pathways delay physiological ovarian aging in women. B. caecimuris, Bacteroides caecimuris; *B. vulgatus* , *Bacteroides vulgatus* ; BAX, BCL2‐associated X protein; β‐RA, β‐resorcylic acid; BSH, bile salt hydrolase; CD38, cluster of differentiation 38; CDCA, chenodeoxycholic acid; FFAR2, free fatty acid receptor 2; FMT, fecal microbiota transplantation; FXR, farnesoid X receptor; GF, germ‐free; GLP‐1, glucagon‐like peptide 1; GPR43 (FFAR2), G protein‐coupled receptor 43 (free fatty acid receptor 2); GATA3, GATA‐binding protein 3; gWAT, gonadal white adipose tissue; HPO, hypothalamic–pituitary–ovarian; IL‐1β, interleukin‐1 beta; IL‐6, interleukin‐6; IL‐22, interleukin‐22; *L. reuteri* , Limosilactobacillus reuteri; *L. salivarius* , Ligilactobacillus salivarius; LPS, lipopolysaccharide; NAD^+^, nicotinamide adenine dinucleotide; NAMPT, nicotinamide phosphoribosyltransferase; Neu5Ac, N‐acetylneuraminic acid; NF‐κB, nuclear factor‐κB; *P. johnsonii* , *Parabacteroides johnsonii* ; PCOS, polycystic ovary syndrome; POI, premature ovarian insufficiency; SCFAs, short‐chain fatty acids; SIRT1, sirtuin 1; SOX7, SRY‐box transcription factor 7; TGR5, G protein‐coupled bile acid receptor 1; TLR4, Toll‐like receptor 4; TNF‐α, tumor necrosis factor alpha; Trp, tryptophan; TUDCA, tauroursodeoxycholic acid.

### 4.1 Gut Ecosystem and Intestinal Barrier: The Local Initiating Layer

For orally administered probiotics, the intestinal ecosystem represents the most plausible initial site of action. Changes in microbial composition or function can modify metabolite production and intestinal barrier properties and, under some conditions, alter host exposure to microbiota‐associated inflammatory molecules. However, this step should not be generalized as a demonstrated effect of every probiotic strain. Relatively strong mechanistic support for a barrier–endotoxin–ovary connection comes from microbiota‐targeted PCOS models. Inulin‐mediated enrichment of SCFA‐producing bacteria was accompanied by improved intestinal barrier integrity, reduced circulating LPS‐binding protein, and attenuation of ovarian TLR4–myeloid differentiation primary response 88 (MyD88)–NF‐κB and NLRP3‐associated inflammatory signaling. Importantly, exogenous LPS administration reversed several of these protective effects, providing intervention‐based support for endotoxin exposure as a mediator between intestinal dysfunction and ovarian inflammation (Geng et al. 2025).

Evidence that an intact microbial ecosystem is required for probiotic‐associated ovarian protection is now emerging from POI models. In a Tripterygium‐glycoside‐induced POI model, *Lactobacillus salivarius* Li01 improved reproductive hormone profiles, follicular indices, and ovarian inflammatory and antioxidant markers in conventionally colonized mice, whereas these protective effects were largely lost under germ‐free conditions (Yao et al. 2026). The germ‐free experiment does not by itself identify a single downstream mediator, but it substantially strengthens the inference that the ovarian phenotype depends on gut microbial context rather than reflecting a gut‐independent systemic action of the administered strain. Thus, intestinal microbial remodeling should be regarded as an upstream mechanistic layer whose contribution must be demonstrated for individual interventions rather than assumed from probiotic identity alone.

### 4.2 Microbiota‐Derived Metabolites as Molecular Relays

SCFAs provide one of the clearest examples of a metabolite relay between the intestinal microbiota and reproductive tissues. In rat primary granulosa cells and human KGN cells, butyrate was sensed through GPR41/GPR43 and increased histone H3 lysine 9 (H3K9) acetylation, with downstream effects on peroxisome proliferator‐activated receptor γ (PPARγ), cluster of differentiation 36 (CD36), and steroidogenic acute regulatory protein (StAR)‐associated steroidogenesis, as well as peroxisome proliferator‐activated receptor γ coactivator 1α (PGC1α)‐mediated mitochondrial dynamics and oxidative homeostasis (Ye et al. 2021). This provides direct cellular evidence that ovarian granulosa cells can respond to SCFA‐receptor signaling, although it does not establish an anti‐aging effect in vivo. Complementary animal evidence indicates that SCFAs can also act indirectly through peri‐ovarian tissues. In sows and mouse experiments, microbiota‐derived SCFAs activated GPR43 in peri‐ovarian gonadal white adipose tissue, increased leptin secretion, reduced granulosa‐cell apoptosis, and promoted follicular maturation (Xu et al. 2025). Together, these findings suggest that SCFAs may signal to the ovary both directly and through extra‐ovarian metabolic intermediates; however, neither model demonstrates that SCFA supplementation delays physiological ovarian aging in women.

Bile acids constitute another major microbiota‐modified signaling system, but their ovarian effects are context‐ and receptor‐dependent. In PCOS, transplantation of fecal microbiota from affected women or colonization with *Bacteroides vulgatus* altered the bile‐acid pool, reduced IL‐22 production, and impaired ovarian function and fertility; administration of selected bile acids or IL‐22 ameliorated several of these abnormalities, supporting a gut microbiota–bile acid–GATA‐binding protein 3 (GATA3)–IL‐22 pathway (Qi et al. 2019). More recently, oral *Bifidobacterium animalis* subsp. lactis BGI‐L99 in a dehydroepiandrosterone (DHEA)‐induced PCOS mouse model restored chenodeoxycholic acid (CDCA) and TUDCA, increased intestinal TGR5/GATA3‐associated signaling and IL‐22, and improved estrous‐cycle, follicular, and reproductive‐endocrine abnormalities. Direct CDCA or tauroursodeoxycholic acid (TUDCA) supplementation reproduced several of the probiotic‐associated phenotypes, strengthening the evidence that bile acids participate in the response (Wang, Chen, et al. 2026). Nevertheless, genetic validation of TGR5 or IL‐22 dependency was not performed, and this mechanism should therefore be described as mechanistically supported rather than definitively established.

FXR‐related signaling further illustrates why bile‐acid pathways should not be treated as uniformly protective. In a PCOS‐like mouse model, the *B. vulgatus* ‐derived metabolite agmatine activated FXR in intestinal enteroendocrine L cells, suppressed glucagon‐like peptide‐1 (GLP‐1) secretion, and promoted insulin resistance and ovarian dysfunction (Yun et al. 2024). A 2026 study identified a separate microbiota‐dependent pathway in which N‐acetylneuraminic acid promoted expansion of a bile‐salt‐hydrolase‐active Ligilactobacillus salivarius, reduced TUDCA availability, enhanced intestinal FXR activation, suppressed IL‐22, and exacerbated ovarian pathology. Importantly, pharmacological FXR blockade and intestine‐specific Fxr deletion attenuated these effects (Zhao et al. 2026). These findings indicate that the biological consequences of microbiota‐modified bile acids depend on metabolite identity, receptor context, and host state; FXR and TGR5 therefore should not be depicted as interchangeable components of a single uniformly beneficial pathway.

Amino‐acid‐derived metabolites may provide additional links between the gut ecosystem and ovarian cellular metabolism. In the Li01 POI study, microbiome and metabolomic analyses implicated altered tryptophan–kynurenine metabolism, while germ‐free experiments established that the ovarian protective phenotype required an intact microbial ecosystem. Li01 combined with tryptophan was associated with increased ovarian nicotinamide adenine dinucleotide (NAD^+^) availability, increased nicotinamide phosphoribosyltransferase (NAMPT), reduced cluster of differentiation 38 (CD38), and sirtuin 1 (SIRT1)‐related changes (Yao et al. 2026). Because several of the strongest downstream NAD^+^ effects were observed under combined Li01‐plus‐tryptophan intervention, the available data support involvement of a microbiota–tryptophan–NAD^+^ axis but do not justify assigning every step of this pathway to Li01 alone.

More direct metabolite‐to‐ovary evidence has been obtained in chemotherapy‐induced POI. Limosilactobacillus reuteri DSM 17938 and β‐resorcylic acid (β‐RA) were reduced in a cisplatin‐induced POI model, whereas supplementation with the strain or β‐RA attenuated hormonal disruption, follicular loss, and ovarian injury. Mechanistic experiments showed that β‐RA suppressed nuclear SOX7 accumulation and downstream BAX‐associated granulosa‐cell apoptosis (Feng et al. 2024). The SOX7/BAX mechanism should therefore be attributed primarily to β‐RA rather than stated as a directly demonstrated molecular action of the live bacterial strain.

A distinct metabolite mechanism has recently been demonstrated in physiological reproductive aging. Young‐donor FMT improved ovulation, oocyte maturation, mitochondrial function, oocyte quality, and fertility‐related outcomes in reproductively aged mice. Multi‐omics and intervention experiments implicated a Bacteroides caecimuris–glutamic acid pathway, and glutamic‐acid supplementation reproduced improvement in aged‐oocyte quality (Wang, Zeng, et al. 2026). This study is particularly relevant because it links gut microbial manipulation to an age‐related reproductive phenotype rather than to an induced PCOS or POI model, although FMT and B. caecimuris are not equivalent to a defined probiotic intervention.

Microbial modification of reproductive steroids represents another potential endocrine interface, but broad “estrobolome” claims require careful qualification. Recent experimental work provides direct evidence that individual gut organisms can alter steroid bioavailability: *Clostridium innocuum* enzymatically converted progesterone to a metabolite with minimal progestogenic activity, lowered circulating progesterone after administration to female mice, and, with prolonged exposure, arrested estrous cyclicity and follicular development (Chen et al. 2024). By contrast, the relationship between predicted microbial β‐glucuronidase activity and circulating estrogen was not straightforward in a recent ovarian‐aging FMT study. Thus, gut microbial steroid biotransformation is biologically relevant, but strain‐specific enzymatic functions should not be generalized into a uniform probiotic‐mediated increase or decrease in estrogen availability.

### 4.3 Systemic Immune, Metabolic, and Endocrine Propagation

Gut‐derived signals need not act directly on ovarian cells to influence reproductive phenotypes. Instead, the systemic layer can integrate microbial signals through immune mediators, metabolic hormones, adipose‐tissue signals, and reproductive endocrine networks. The LPS–TLR4–NF‐κB pathway illustrates an inflammatory route (He et al. 2022), whereas bile‐acid‐dependent modulation of IL‐22 provides an immune relay and SCFA–GPR43‐dependent leptin secretion from peri‐ovarian adipose tissue provides a metabolic relay (Qi et al. 2019; Wang, Chen, et al. 2026). Other microbiota‐derived signals can influence GLP‐1 and insulin sensitivity through intestinal FXR (Yun et al. 2024; Zhao et al. 2026). These pathways converge systemically before affecting ovarian inflammation, steroidogenesis, follicular development, or cell survival. Consequently, an improvement in ovarian phenotype after a probiotic intervention does not necessarily imply direct probiotic targeting of the ovary; the effect may be mediated through broader metabolic or immune normalization.

Limited human observations are also compatible with systemic endocrine modulation. For example, an exploratory *Bifidobacterium lactis* V9 intervention in women with PCOS reported heterogeneous changes in reproductive hormones and intestinal SCFAs, illustrating potential host‐dependent microbiota–endocrine interactions rather than demonstrating direct restoration of the hypothalamic–pituitary–ovarian (HPO) axis (Zhang et al. 2019).

### 4.4 Ovarian Convergence: Redox Homeostasis, Cell Survival, and Reproductive Function

The intestinal and systemic signals described above ultimately converge on the ovarian processes outlined in Section 2. SCFA signaling provides examples of both direct granulosa‐cell regulation of mitochondrial and steroidogenic pathways and indirect regulation through peri‐ovarian adipose tissue. β‐RA provides more specific evidence for modulation of granulosa‐cell apoptosis through SOX7/BAX‐associated signaling in chemotherapy‐induced POI, whereas the B. caecimuris–glutamic acid pathway links gut microbial remodeling to mitochondrial function and oocyte quality in advanced reproductive age. Li01‐associated protection in POI involves follicular, inflammatory, and antioxidant ovarian endpoints but remains dependent on the surrounding gut microbial ecosystem. More recently, oral *Parabacteroides johnsonii* administration in middle‐aged mice improved ovarian reserve, reduced follicular atresia, enhanced granulosa‐cell proliferation, and decreased systemic inflammation, providing defined‐species preclinical evidence directly relevant to age‐associated ovarian functional decline (Wang, Zeng, et al. 2026). These findings collectively support the concept that ovarian redox state, mitochondrial competence, granulosa‐cell survival, steroidogenesis, follicular development, and oocyte quality can serve as downstream outcomes of gut‐derived signaling.

However, the proximity of evidence to physiological ovarian aging differs substantially among these endpoints. The *L. reuteri* /β‐RA and Li01 studies were conducted in experimentally induced POI, while the bile‐acid/IL‐22 and FXR studies largely concern PCOS. In contrast, young‐donor FMT and glutamic‐acid experiments directly addressed advanced reproductive age and aged‐oocyte quality. Therefore, improvement of granulosa‐cell apoptosis, oxidative markers, or reproductive hormones in a disease or toxicant model should not be interpreted as equivalent to slowing the physiological depletion of the human ovarian reserve.

### 4.5 Causal Evidence, Bidirectionality, and Limits of Mechanistic Inference

The mechanistic literature can be interpreted using an evidence hierarchy. At the lowest level, association studies identify correlations between microbial taxa or metabolites and ovarian phenotypes. Intervention‐level evidence is stronger when probiotic administration or FMT modifies both the microbiota and reproductive outcomes. Mechanistic mediation requires additional perturbation, such as loss of efficacy in germ‐free animals, metabolite rescue or supplementation, receptor blockade or genetic deletion, or transfer of a defined microbial community. These levels reflect increasing confidence in causal mechanism, but they should not be interpreted as equivalent levels of clinical translatability.

The gut–ovarian axis should also be considered bidirectional rather than a unidirectional pathway in which aging simply causes dysbiosis and dysbiosis subsequently damages the ovary. Natural reproductive aging and experimentally induced ovarian failure are associated with changes in gut microbial profiles, supporting the possibility that ovarian endocrine state can itself reshape the intestinal ecosystem. At the same time, FMT experiments demonstrate that altered microbial communities can modify ovarian phenotypes. Notably, a 2026 study found that transplantation of microbiota from estropausal donors into young adult mice unexpectedly reduced ovarian inflammatory gene expression and improved several measures of ovarian health rather than reproducing an “aged” ovarian phenotype (Kim et al. 2026). This finding argues against a simplistic classification of age‐associated microbiota as uniformly detrimental and raises the possibility that some late‐life microbial changes reflect compensatory adaptations rather than direct drivers of ovarian decline.

Taken together, the mechanistic literature supports several model‐specific routes by which gut‐derived signals can influence ovarian phenotypes, but the translational weight of these pathways differs substantially across experimental systems. Their relevance to human reproductive aging therefore requires separate consideration of clinical evidence, administration route, dose feasibility, product characteristics, and safety, as discussed below.

## 5 Translational Relevance, Human Evidence, Safety, and Current Limitations

### 5.1 Human Evidence and Limits of Disease‐Model Extrapolation

Translation of the gut–ovarian axis from experimental models to human reproductive aging requires a clear distinction between evidence of biological plausibility, evidence of benefit in related ovarian disorders, and direct evidence that an intervention modifies ovarian aging itself. Direct defined‐strain evidence in POI and physiological reproductive‐aging models remains predominantly preclinical. In contrast, a substantial human randomized‐trial literature now exists in PCOS. Consistent with the prespecified evidence‐gap criteria in Table S1, we distinguished direct ovarian‐reserve endpoints from indirect metabolic, endocrine, symptom, and quality‐of‐life outcomes, and further distinguished short‐term changes in ovarian‐reserve biomarkers from longitudinal evidence capable of testing whether the rate of physiological ovarian‐reserve decline is altered.

Two recent meta‐analyses have synthesized microbiota‐targeted supplementation in PCOS, but they should not be interpreted as independent bodies of evidence because their primary‐study sets overlap. Sun et al. (2026) synthesized 11 RCT reports, comprising 780 participants as reported in the review, and reported statistically significant pooled changes in fasting insulin (MD −1.41, 95% CI −2.76 to −0.05; *I* ^2^ = 0%), total testosterone (MD −0.19, 95% CI −0.25 to −0.12; *I* ^2^ = 46%), triglycerides (MD −12.28, 95% CI −24.21 to −0.35; *I* ^2^ = 0%), and low‐density lipoprotein cholesterol (MD −6.19, 95% CI −10.08 to −2.30; I^2^ = 31%) (Sun et al. 2026). Yin et al. (2026) included a broader set of 26 randomized trials of prebiotics, probiotics, and synbiotics and also reported statistically significant pooled changes in several metabolic and endocrine outcomes; however, heterogeneity was substantially higher for fasting insulin (SMD −0.60, 95% CI −1.12 to −0.08; *I* ^2^ = 85.1%) and testosterone (SMD −1.47, 95% CI −2.32 to −0.62; *I* ^2^ = 92.1%). In the relevant synbiotic subgroup analyses, triglycerides (SMD −0.59, 95% CI −1.11 to −0.07; *I* ^2^ = 83.6%) and LDL cholesterol (SMD −0.62, 95% CI −1.16 to −0.09; *I* ^2^ = 84.5%) also showed statistically significant pooled changes (Yin et al. 2026). The corresponding pooled effect estimates, 95% confidence intervals, and heterogeneity statistics are summarized in Table S3, Panel B.

Trial‐level cross‐matching showed that 9 of the 11 RCT reports included by Sun et al. were also represented in Yin et al. However, two pairs of reports in Sun et al. arose from the same parent trials; after collapsing multiple publications from the same participant cohort, the 11 reports represented 9 unique parent trials/cohorts, 7 of which were also represented in Yin et al. (Table S3, Panel A). The two meta‐analyses should therefore be regarded as substantially overlapping syntheses rather than independent replications. Their pooled estimates should not be combined or compared as if they represented separate evidence bases. Moreover, the effect metrics, intervention scopes, and primary‐study compositions differed between the reviews, and several pooled estimates in the broader analysis showed substantial heterogeneity. The clinical importance of these statistically significant biomarker changes remains uncertain because neither meta‐analysis prespecified outcome‐specific minimally important differences for these selected endpoints. The evaluated outcomes predominantly represent metabolic, androgen‐related, lipid, and oxidative‐stress features of PCOS rather than longitudinal measures of ovarian‐reserve loss; PCOS should therefore not be used as a surrogate for physiological reproductive aging.

Microbiome–reproductive interactions are not limited to the intestinal compartment. Vaginal and reproductive‐tract microbial communities have also been associated with infertility and other reproductive outcomes, and local probiotic interventions may alter vaginal microbial composition (Lin et al. 2024). However, these studies interrogate a distinct microbial niche and currently provide little direct evidence regarding ovarian reserve or physiological ovarian aging. Vaginal‐microbiota findings should therefore be considered complementary reproductive‐microbiome evidence rather than direct evidence for the gut–ovarian axis.

Human evidence closer to the menopausal transition is emerging but likewise does not yet demonstrate preservation of the ovarian reserve. In a randomized trial, *Lactobacillus acidophilus* YT1 improved menopausal symptom and quality‐of‐life scores without significant between‐group changes in FSH or estradiol (Lim et al. 2020). An exploratory randomized, double‐blind, placebo‐controlled trial of a three‐strain formulation containing Levilactobacillus brevis KABP052, Lactiplantibacillus plantarum KABP051, and *Pediococcus acidilactici* KABP021 randomized 115 healthy peri‐ or postmenopausal women, with 111 included in the per‐protocol analysis. The trial was prospectively registered (UMIN000045547) and reported maintenance of circulating estrone and estradiol over 12 weeks, although menopausal symptom scores did not differ significantly between groups and neither gut microbial composition nor ovarian‐reserve endpoints were assessed (Honda et al. 2024). The study reported industry funding and involvement of industry‐affiliated investigators.

A subsequent randomized, double‐blind, placebo‐controlled trial evaluated the same KABP052/KABP051/KABP021 formulation in 245 peri‐ or postmenopausal women and was registered as NCT06604234. This study reported modest improvements in menopause‐related symptom scores and selected quality‐of‐life measures but did not assess circulating sex hormones, gut microbiota, AMH, AFC, or other ovarian‐reserve endpoints (Rodríguez‐Palmero et al. 2026). Honda et al. and Rodríguez‐Palmero et al. should not be interpreted as fully independent replications: they evaluated the same three‐strain formulation, involved overlapping industry‐affiliated investigators, and both reported industry sponsorship. The later trial therefore extends the clinical dataset for the same commercial formulation rather than constituting an independent replication by a separate product and investigator group.

Detailed study‐level characteristics, including sample size, randomization, blinding, comparator, prospective registration, attrition, funding/sponsorship, and product/investigator overlap, are summarized in Table S2. Methodological appraisal of these individual human intervention studies further tempers the strength of the clinical evidence. Study designs range from very small uncontrolled cohorts to randomized, double‐blind, placebo‐controlled trials, and direct ovarian‐aging endpoints are uncommon. For example, the *Bifidobacterium lactis* V9 PCOS study included only 14 women completing the probiotic phase and had no concurrent placebo group (Zhang et al. 2019), whereas the study by Ji et al. randomized 60 women among probiotic, metformin, and combination groups but did not include a true placebo arm (Ji et al. 2022). The 6‐month PCOS study by Kaur et al. randomized 104 women and included a placebo comparison, but evaluated a multi‐strain probiotic/FOS formulation alongside standardized lifestyle advice, limiting attribution to an individual strain or microbial component (Kaur et al. 2022). The menopause‐related trials by Lim et al., Honda et al., and Rodríguez‐Palmero et al. used randomized placebo‐controlled designs, but their principal outcomes were symptoms, quality of life, or circulating endocrine measures rather than longitudinal ovarian‐reserve decline. Across the human evidence base, prospective registration, attrition, comparator design, blinding, and funding/sponsorship are therefore reported study by study in Table S2, with items recorded as not reported when they could not be verified from the primary publication.

A further randomized controlled trial evaluated Limosilactobacillus reuteri NCU‐37 in infertile women who developed leuprorelin‐induced perimenopausal syndrome during assisted reproductive treatment. Four weeks of oral supplementation was associated with improved symptom scores and higher anti‐Müllerian hormone (AMH), estradiol, FSH, and LH levels relative to placebo, together with alterations in the fecal microbiota (Wu et al. 2026). This study provides clinically relevant reproductive‐endocrine evidence, but the model represents reversible pharmacological suppression of the hypothalamic–pituitary–ovarian axis during assisted reproduction rather than natural perimenopause or spontaneous POI. Short‐term changes in AMH under this setting should therefore not be interpreted as evidence of a slower rate of physiological follicular depletion. Its short 4‐week intervention period and iatrogenic reproductive context further limit inference regarding durable preservation of the follicular reserve. In our structured literature and trial‐registry searches, we did not identify a completed randomized human trial specifically designed to test whether probiotic supplementation slows the longitudinal physiological loss of ovarian reserve or alters the natural trajectory of reproductive aging. The registry audit identified studies in POI, DOR, and related ovarian‐dysfunction settings that include AMH, AFC, or other ovarian‐function outcomes; however, these studies address disease‐specific or short‐term ovarian endpoints rather than the longitudinal rate of physiological age‐related follicular depletion. Human evidence should therefore be described as disease‐ or menopause‐related translational evidence rather than proof of an ovarian anti‐aging effect. Taken together, the human intervention evidence is heterogeneous in methodological rigor, clinical context, product independence, and endpoint relevance. Randomized, blinded, placebo‐controlled designs are available for several preparations, but many studies remain modest in size, relatively short in duration, focused on disease‐specific or symptom‐related outcomes, or subject to product/investigator overlap. These limitations do not invalidate the reported clinical signals, but they substantially restrict inference about preservation of ovarian reserve or modification of physiological reproductive aging. Future studies should therefore prioritize adequately powered, prospectively registered, randomized, double‐blind, placebo‐controlled trials of well‐characterized probiotic strains in clearly defined reproductive‐aging‐ or POI‐relevant populations, with prespecified ovarian endpoints, transparent attrition reporting, dose justification, longitudinal follow‐up, independent replication, and standardized safety assessment.

### 5.2 Administration Route and Relevance to Functional‐Food Translation

Administration route is an important determinant of translational relevance. Oral administration as a food, capsule, powder, sachet, or other dietary formulation is most directly aligned with functional‐food development. Oral gavage in animal models also establishes gastrointestinal exposure, but it delivers a controlled bolus under experimental conditions and should not be treated as equivalent to voluntary dietary intake in humans. Fecal microbiota transplantation is valuable for testing whether a microbial ecosystem can causally transfer or modify a reproductive phenotype, but an undefined fecal community is not equivalent to a defined probiotic strain or nutritional product. Local uterine or reproductive‐tract administration can provide useful mechanistic information on host–microbe interactions but does not constitute a conventional functional‐food route. Similarly, intraperitoneal administration of microbial metabolites or other compounds can test systemic biological activity but should not be used as the basis for direct probiotic‐food dose extrapolation. Route‐specific evidence should therefore be identified explicitly when assessing translational potential.

### 5.3 Dose Translation and Formulation

Probiotic dose translation should not be based on a simple linear conversion of animal colony‐forming units (CFU) per kilogram to human body weight. Conventional body‐surface‐area methods for estimating a human‐equivalent dose were developed primarily to derive safe starting exposures for therapeutic agents from animal toxicology data, rather than to establish the efficacy‐equivalent dose of a living microbial product. For probiotics, CFU quantifies viable organisms rather than a systemically absorbed chemical exposure, and biological activity depends on strain identity, formulation, viability during storage and gastrointestinal transit, food matrix, administration frequency, treatment duration, and host context. Accordingly, an animal dose such as 10^9^ CFU/kg should be reported transparently in its original experimental form rather than converted into an apparently precise “human‐equivalent CFU.” Human development should instead be anchored to strain‐specific clinical dose‐ranging and to doses that have already demonstrated feasibility in relevant populations.

Existing reproductive‐health trials illustrate why clinically observed doses are more informative than theoretical interspecies conversion. MENOLACTO YT1 was administered at 1 × 10^8^ CFU/day for 12 weeks (Lim et al. 2020), the KABP052/KABP051/KABP021 formula at approximately 1 × 10^9^ CFU/day for 12 weeks or 3 months (Honda et al. 2024; Rodríguez‐Palmero et al. 2026), NCU‐37 at 4 × 10^9^ CFU/day for 4 weeks (Wu et al. 2026), and a seven‐strain PCOS formulation at approximately 1 × 10^10^ CFU/day for 6 months (Kaur et al. 2022). These values should not be interpreted as a universal therapeutic range; rather, they demonstrate that dose, strain composition, formulation, and treatment duration need to be validated together for each intended indication.

### 5.4 Safety, Strain Identity, and Product Quality

The potential suitability of probiotics for long‐term nutritional intervention should not be equated with unconditional safety. Moreover, probiotic properties and documented health effects should be interpreted at the level of adequately characterized strains and formulations rather than generalized across the entire probiotic category (Hill et al. 2014). Safety assessment should be strain‐ and product‐specific and should include unambiguous taxonomic identification, screening for acquired antimicrobial‐resistance determinants and relevant virulence traits, manufacturing purity, viable‐count consistency, storage stability, and systematic adverse‐event reporting. The European Food Safety Authority (EFSA) Qualified Presumption of Safety (QPS) framework illustrates this principle: even for taxonomic units considered suitable for QPS assessment, relevant qualifications remain strain‐specific, and bacterial strains should not harbor acquired resistance genes to therapeutically important antimicrobials (Hazards, E. P. o. B., et al. 2026). Likewise, regulatory guidance for live biotherapeutic products emphasizes characterization and manufacturing controls rather than treating all microorganisms within a nominal probiotic category as interchangeable.

Invasive infections associated with organisms used as probiotics appear uncommon in the general population, although risk is not uniform across host populations. Case reports demonstrate that opportunistic infection can occur in individuals with severe underlying disease or major disruption of host barriers; for example, Lacticaseibacillus paracasei bacteremia and mediastinitis were reported in a patient with invasive thoracic malignancy and esophageal involvement (Kushima et al. 2026). Such observations should not be extrapolated to imply substantial infection risk in healthy consumers, but they support additional caution in severely immunocompromised individuals and in patients with conditions predisposing to microbial translocation. Reproductive‐health trials should therefore prespecify exclusion or monitoring criteria for clinically vulnerable participants and should report adverse events rather than assuming safety from historical food use alone. Pregnancy should likewise be handled as a population‐specific safety consideration rather than through a blanket assumption of either safety or contraindication; available pregnancy studies are reassuring for several tested formulations, but these findings cannot automatically be generalized across strains and products (Sheyholislami and Connor 2021).

### 5.5 Regulatory Considerations

Regulatory classification also affects the translational pathway. In the United States, the US Food and Drug Administration (FDA) does not recognize “probiotic” as a single regulatory product category; depending on intended use and claims, a live microbial product may be regulated as a conventional food, dietary supplement, drug, or biological product. Dietary supplements are not pre‐approved by the FDA for efficacy before marketing, although manufacturers remain responsible for compliance with applicable safety, labeling, and quality requirements. A live microbial product developed for therapeutic use may instead enter a drug/live‐biotherapeutic regulatory pathway involving additional clinical and manufacturing requirements. In the European Union, health claims made for foods are governed by Regulation (EC) No 1924/2006 and must be scientifically substantiated and comply with the applicable authorization requirements. Regulatory strategy should therefore be considered early in product development, particularly because a claim to “delay ovarian aging” or “treat POI” carries substantially different evidentiary implications from a general nutritional or wellbeing claim. These findings provide a rationale for evaluating well‐characterized probiotic formulations in reproductive‐health applications, while any ovarian‐aging‐specific health or therapeutic claim will require indication‐specific clinical substantiation and compliance with the applicable regulatory framework.

### 5.6 Critical Interpretation and Priorities for Clinical Translation

A further limitation is that an improved ovarian phenotype after probiotic administration does not establish direct ovarian targeting. Improvements in insulin sensitivity, adipose‐tissue signaling, systemic inflammation, intestinal barrier function, or other aspects of host physiology may secondarily modify endocrine or ovarian endpoints. Such indirect effects remain biologically important, but they should be distinguished from evidence that a microbial strain or metabolite reaches the ovary and acts directly on ovarian cells. The mechanistic strength of a study therefore depends not only on whether the ovarian phenotype improves, but also on whether the proposed mediator has been tested by rescue, receptor manipulation, genetic perturbation, germ‐free conditions, metabolite supplementation, or related causal approaches.

Future trials intended to test an ovarian‐aging or POI‐related claim should be designed around the clinical question rather than around generic probiotic endpoints. Depending on the intended indication, appropriate populations may include women undergoing the natural menopausal transition or carefully defined POI/diminished‐ovarian‐reserve populations. Primary outcomes should be prespecified and should distinguish ovarian‐reserve biomarkers from evidence of altered reproductive‐aging trajectories. AMH and antral follicle count may provide reserve‐related information, while serial changes in these measures, FSH/E2 profiles, menstrual and ovulatory function, and, where clinically appropriate, oocyte, embryo, pregnancy, or live‐birth outcomes address different levels of reproductive function. Trials should use taxonomically and genomically characterized oral strains, clinically feasible formulations, verified viable counts throughout shelf life, appropriate placebo controls, dietary and medication documentation, adherence monitoring, and standardized adverse‐event surveillance. Prespecified mechanistic secondary outcomes may include circulating LPS and inflammatory cytokines, SCFAs, bile acids, tryptophan‐related metabolites, endocrine and metabolic markers, and strain‐level microbiome changes. Such measurements are most informative when incorporated prospectively to test mediation rather than used post hoc to infer an ovarian anti‐aging mechanism.

Accordingly, the current evidence supports a biologically plausible and clinically testable gut–ovarian axis, but the evidentiary threshold for demonstrating modulation of ovarian physiology is lower than that required to claim delayed physiological ovarian aging. Maintaining this distinction is essential for both scientific interpretation and functional‐food translation.

## 6 Conclusions and Future Priorities

Collectively, current evidence supports the gut–ovarian axis as a biologically plausible and experimentally testable framework through which intestinal microbial ecology, barrier function, and microbiota‐derived metabolites may influence systemic immune, metabolic, and endocrine signaling relevant to ovarian physiology. Selected probiotic and microbiota‐directed interventions have improved ovarian or reproductive phenotypes in experimental POI, chemotherapy‐induced ovarian injury, PCOS, and reproductive‐aging models, with stronger mechanistic support in studies incorporating germ‐free conditions, microbiota transplantation, metabolite supplementation, receptor manipulation, or related causal approaches. However, these effects are highly strain‐, model‐, dose‐, route‐, and context‐dependent, and improvement of an ovarian phenotype in an experimental model does not establish delayed physiological ovarian aging in women.

An important qualification concerns the directionality of microbiota–ovary effects. In young adult recipients, FMT from estropausal donors unexpectedly reduced ovarian inflammatory gene expression and improved several measures of ovarian health rather than inducing an aged ovarian phenotype (Kim et al. 2026). This discordant finding argues against treating age‐associated microbiota as uniformly detrimental and suggests that some late‐life microbial changes may be compensatory or context dependent rather than direct drivers of ovarian decline. Because the recipients were young adults, however, these findings should not be interpreted as evidence that estropausal‐donor microbiota rejuvenate an aged ovary. More broadly, microbiota‐targeted strategies should therefore be evaluated according to defined microbial functions, host context, and validated ovarian outcomes rather than a simple objective of restoring a “younger” microbiota.

Human translation therefore represents the principal unresolved step. Existing human studies demonstrate that selected probiotic interventions can modify metabolic, inflammatory, endocrine, menstrual, symptom, or fertility‐related outcomes in related reproductive settings. However, our structured literature and trial‐registry searches did not identify a completed randomized human trial specifically designed to demonstrate a reduced longitudinal rate of physiological ovarian‐reserve decline or delayed physiological ovarian aging.

Importantly, the registry audit identified work in progress that moves closer to direct ovarian endpoints. Registered studies in POI or ovarian dysfunction (NCT06866990 and NCT07308327) include AMH, AFC, or related ovarian‐function outcomes, while ChiCTR2600116322 evaluates an intervention in women with diminished ovarian reserve and includes AMH and AFC as registered outcomes. These studies may strengthen the evidence base for microbiota‐targeted modulation of ovarian function when results become available; however, their disease‐focused populations, short‐term endpoints, and/or co‐interventions mean that they do not directly test whether probiotics slow the physiological age‐related decline in ovarian reserve.

The next priority is consequently not simply to conduct “more probiotic trials” but to test well‐characterized, orally feasible strains in clearly defined ovarian‐aging‐ or POI‐relevant populations using prespecified ovarian endpoints, longitudinal assessment where an anti‐aging claim is intended, mechanistic microbiome/metabolite biomarkers, rigorous product characterization, and standardized safety monitoring. Until such evidence is available, probiotic‐based functional foods should be regarded as promising microbiota‐targeted candidates for female reproductive‐health research rather than established interventions for extending reproductive lifespan or delaying physiological ovarian aging.

## Author Contributions

**Meiqi Chen:** writing – original draft, writing – review and editing, visualization. **Qi Su:** validation. **Mengshuang Sun:** validation. **He Zhang:** software. **Jin Xu:** data curation. **Chenxi Zhang:** validation. **Yanbo Wang:** conceptualization, funding acquisition, supervision.

## Funding

This work was supported by the National Natural Science Foundation of China (32360182). The Inner Mongolia Autonomous Region Young Scientific and Technological Talents Support Program (NJYT24053). The Inner Mongolia Natural Science Foundation (2026MS0707). The Central Government Guiding Local Science and Technology Development Funds Project (2023ZY0015). The Fundamental Research Funds for the Universities Directly under the Inner Mongolia Autonomous Region (GXKY26Z068). The Inner Mongolia Autonomous Region Postgraduate Education and Teaching Reform Project (JG2025019Z).

## Ethics Statement

The authors have nothing to report.

## Consent

The authors have nothing to report.

## Conflicts of Interest

The authors declare no conflicts of interest.

## Supporting information


**Table S1:** Eligibility criteria for verification of the clinical evidence gap.
**Data S1:** Structured literature‐search strategies and record flow, focused human evidence‐gap screening, clinical‐trial registry audit, publication checks, and 2026 reference‐date audit.
**Table S2:** Methodological appraisal and endpoint classification of human intervention studies.
**Table S3:** Primary‐study overlap, selected pooled effect estimates, and heterogeneity in the PCOS meta‐analyses by Sun et al. (2026) and Yin et al. (2026).

## Data Availability

All data supporting this review are contained within the article and its Supporting Information. No original participant‐level or experimental datasets were generated.
